# Intermittent Pneumatic Impulse Compression in the Treatment of Stasis Dermatitis—A Monocenter Randomized Controlled Trial

**DOI:** 10.3390/jcm14103321

**Published:** 2025-05-09

**Authors:** Sarah Janßen, Julia Schmölders, Theresa Maria Jansen, Neslihan Ertas, Julian-Dario Rembe, Bernhard Homey, Norman-Philipp Hoff

**Affiliations:** 1 Department of Dermatology, Medical Faculty and University Hospital Düsseldorf, Heinrich-Heine-University, 40225 Düsseldorf, Germany; sarah.janssen@med.uni-duesseldorf.de (S.J.); julia.schmoelders@hhu.de (J.S.); bernhard.homey@med.uni-duesseldorf.de (B.H.); 2 Department for Vascular and Endovascular Surgery, Medical Faculty and University Hospital Düsseldorf, Heinrich-Heine-University, 40225 Düsseldorf, Germany; neslihan.ertas@med.uni-duesseldorf.de (N.E.); julian-dario.rembe@med.uni-duesseldorf.de (J.-D.R.)

**Keywords:** intermittent pneumatic compression, stasis dermatitis, dermatology, chronic venous insufficiency, edema, compression, monocenter randomized controlled trial, chronic wounds

## Abstract

**Background/Objectives:** Intermittent pneumatic impulse compression (IIC) is a well-established drainage treatment that reduces edema and enhances arterial blood flow. While widely utilized in various medical fields, its efficacy in dermatology, particularly for stasis dermatitis, remains underexplored. This study evaluates the effectiveness of IIC in inpatients with bilateral stasis dermatitis by comparing standard therapy alone versus standard therapy with additional IIC on one leg over five days. **Methods**: Seventeen patients from the Dermatology Department at University Hospital Duesseldorf were enrolled. Both legs received standard therapy, while one randomized leg received additional IIC for four hours daily. Measurements, including transcutaneous oxygen pressure (tcpO_2_), leg circumference, and pain sensation, were taken at baseline, the first day post-IIC, and after five days. Statistical analysis included paired *t*-tests, with significance set at *p* < 0.05. **Results**: The IIC-treated legs exhibited significant improvements in tissue oxygen saturation (MD = 19.87 mmHg, SD = 27.82, *p* = 0.012) and reduced ankle circumference (MD = −2.125 cm, SD = 1.593, *p* < 0.0001). No significant changes were observed in tcpO_2_ or circumference in the non-IIC-treated legs. Other leg circumference measurements (calf, above the knee) did not demonstrate significant improvements in either group. Pain levels remained stable under IIC therapy. **Conclusions**: This proof-of-concept study provides evidence supporting IIC as a beneficial adjunct therapy for stasis dermatitis, demonstrating significant edema reduction and enhanced oxygenation. Further investigations are warranted to confirm these findings and expand clinical applicability.

## 1. Introduction

Intermittent pneumatic impulse compression (IIC) therapy is an innovative treatment approach that has demonstrated potential across various medical fields in enhancing venous return, lymphatic drainage, and arterial blood flow [1,2]. Most current publications focus on the prevention of embolic events and thrombosis prophylaxis in the perioperative setting [3,4]. Unlike traditional intermittent pneumatic compression, IIC delivers rapid, pulsed pressure bursts within each cycle, more closely simulating the natural muscle pump action. This mechanism helps to mitigate tissue swelling and improves overall recovery by modulating the immune response and promoting better fluid balance in the interstitial space. The rhythmic pressure application increases vascular shear stress, stimulating nitric oxide (NO) release, promoting vasodilation, and improving microcirculation [5]. Additionally, IIC has been shown to reduce inflammation by decreasing inflammatory cell accumulation and cytokine release. These effects make IIC a potentially valuable therapeutic approach for conditions characterized by poor circulation and inflammation, such as stasis dermatitis.

Indications for IIC therapy include the following:

1. Chronic venous insufficiency;
2. Lymphedema;
3. Peripheral arterial disease;
4. Postoperative recovery;
5. Sports medicine and injury rehabilitation.

Contraindications for IIC therapy include the following:

1. Acute untreated infections (e.g., phlebitis, erysipelas);
2. Current thrombosis or pulmonary embolism;
3. Acute decompensated heart failure;
4. Severe peripheral arterial disease;
5. Active bleeding or high risk of hemorrhage.

Stasis dermatitis, a common inflammatory skin condition affecting the lower extremities, is primarily caused by chronic venous insufficiency (CVI). It affects approximately 6% of individuals over 50 years old in the United States, rendering it twice as common as psoriasis [6]. It is characterized by pruritus, pain, edema, and skin discoloration, significantly impacting patients’ quality of life. The condition’s pathophysiology involves venous hypertension, leading to inflammatory cell accumulation and extravasation across the vascular endothelium. The condition manifests as erythema, scaling, pruritus, and hyperpigmentation and can progress to dermatoliposclerosis and venous ulcers [7]. Management primarily targets underlying CVI through compression therapy, leg elevation, and lifestyle modifications [8,9]. However, adherence to compression therapy is often poor due to discomfort, application difficulty, and cost [10]. While topical corticosteroids remain the primary pharmacological treatment, there is a growing need for additional therapeutic options [9,11,12].

Given the limitations of current treatment options and the potential benefits of IIC, this study aims to investigate the efficacy of IIC as an adjunct to standard therapy in patients with stasis dermatitis. We hypothesize that IIC, combined with compression bandages, will enhance blood circulation, improve microcirculation, and reduce edema more effectively than standard therapy alone. To assess the efficacy of IIC, we conducted a prospective randomized study in our dermatology department, performing a side-by-side comparison of legs treated with standard care versus additional IIC over five days. Our study explores IIC’s safety, feasibility, decongestive effects, and potential adverse effects, including pain, in a clinical setting. To our knowledge, this is the first study to investigate the use of IIC specifically for stasis dermatitis management.

## 2. Materials and Methods

### 2.1. Study Design

**Figure 1:**
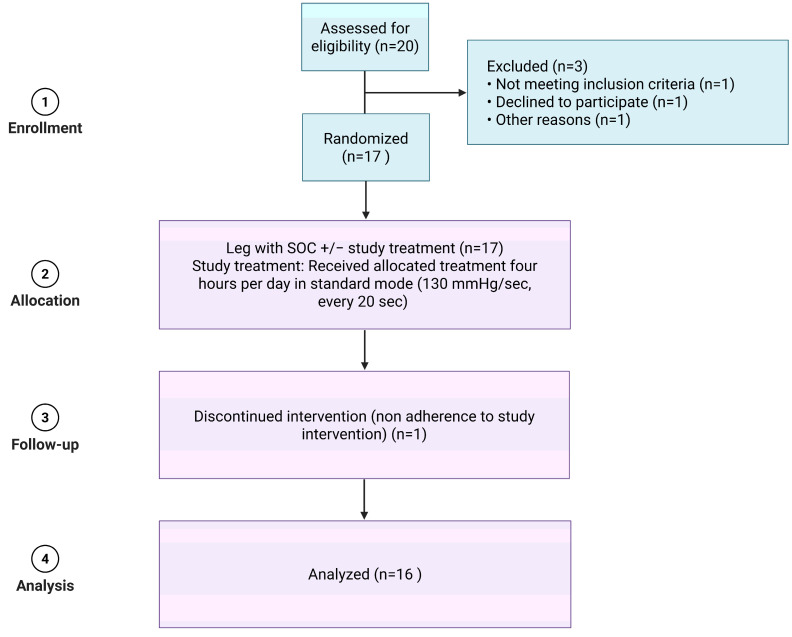
Study design. This figure presents the inclusion and follow-up of the monocenter study. Created in BioRender on 8 May 2025. Homey, B. (2025) https://app.biorender.com/citation/67ca9ba3c60de3d5f48b18f8.

This study is a randomized, controlled, prospective single-blinded monocenter trial to evaluate the efficacy of IIC in patients with stasis dermatitis regarding edema reduction, microcirculatory oxygen supply, and pain sensation compared to standard therapy (Figure 1). This study was conducted in accordance with the Declaration of Helsinki, and a priori ethical approval was obtained 1 April 2021 from the Ethics Committee of the Heinrich Heine University Dusseldorf (number 2021-1386). Patients did not receive any form of compensation for participation and signed informed consent was obtained prior to study initiation.

### 2.2. Definition of Stasis Dermatitis

**Figure 2:**
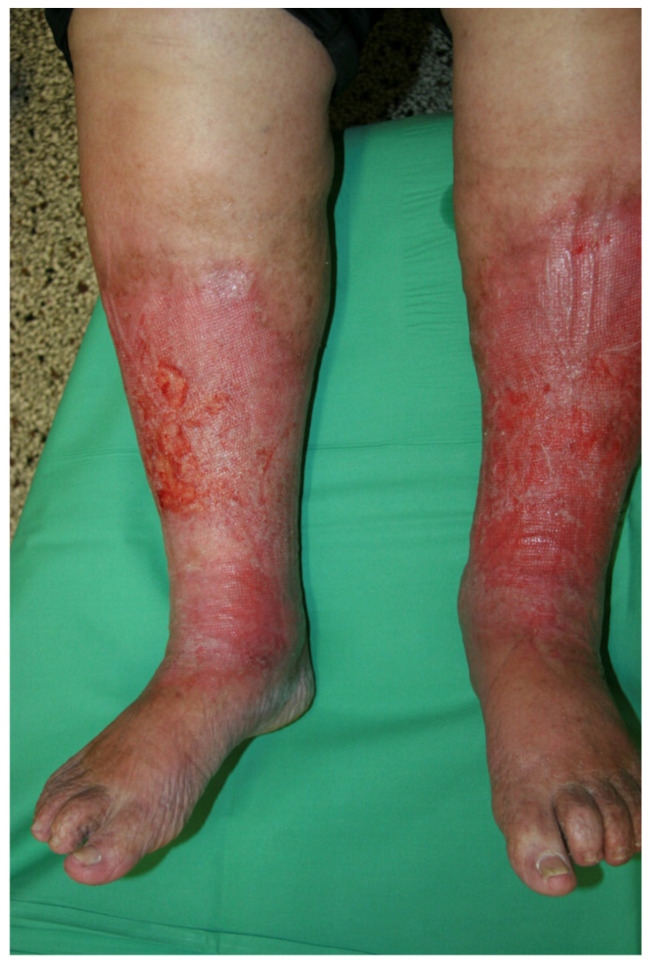
Admission findings of a patient presenting with stasis dermatitis. The bilateral lower extremities exhibit sharply to irregularly demarcated, symmetrical erythema encompassing the entire circumference of the legs. Erosive cutaneous lesions are present on the right and left pretibial regions, indicative of compromised skin barrier integrity. The pedal regions demonstrate comparatively less pronounced dermatological manifestations.

Stasis dermatitis was diagnosed by dermatology specialists who were not involved in the study. The diagnosis was established if the following dermatological parameters were present: bilateral erythema, scaling, and swelling of both lower legs. Pruritus and hyperpigmentation, as well as ulceration, could also be present (Figure 2). Patients with additional erysipelas were pre-treated with anti-infective therapy before being included in the study and treated with impulse compression.

### 2.3. Inclusion and Exclusion Criteria

Recruitment of patients with bilateral stasis dermatitis was conducted at the Department of Dermatology at Dusseldorf University Hospital.

Patients with stasis dermatitis are treated under full inpatient conditions. In the clinical algorithm, patients with stasis dermatitis are hospitalized for an average of 5 days. Therefore, the study intervention was planned for 5 days in the inpatient setting.

**Figure 3:**
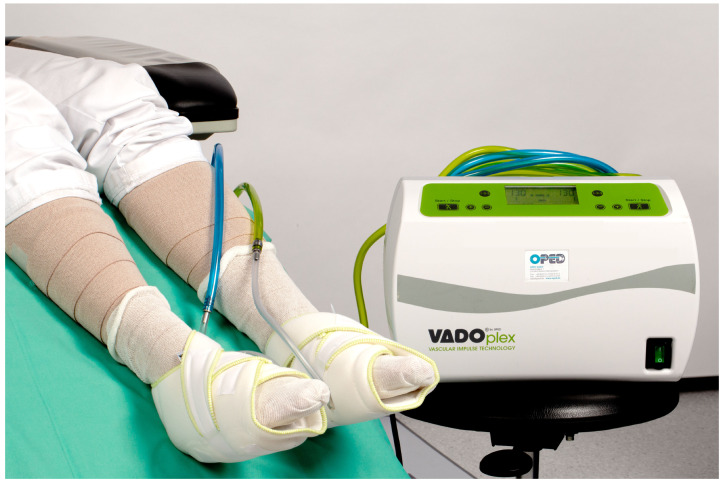
Treatment of a patient using IIC. The subjects were administered compression bandages on both legs to address the stasis dermatitis.

Inclusion and exclusion criteria for the study are summarized in Table 1. After inclusion in the study, the leg to be treated was randomized by random allocation by sealed envelopes. The patients were treated for five days on one leg with IIC using VADOplex^®^ (OPED GmbH, Valley, Germany) for four hours per day in standard mode (130 mmHg/s, every 20 s). Standard therapy (Figure 3) was performed on both legs. The standard therapy consists of topical glucocorticosteroids, elastocompressive bandages, and elevation of the legs.

### 2.4. Standard of Care (SOC)

According to the current standard, patients received treatment on the day of admission, commencing with the application of topical glucocorticosteroids (mometasone furoate or betamethasone) to the affected erythematous area. Following absorption of the cream into the skin (10 min), tubular bandages were applied directly over the skin. Subsequently, a layer of medical cotton wool (Lohmann & Rauscher^®^, Regensdorf (Germany), Vienna (Austria)) was applied. Trained wound specialists then applied short-stretch compression bandages to the legs to ensure effective decongestion. The bandages were replaced daily throughout the duration of hospitalization.

### 2.5. Outcome Parameters

During the study period, measurements including transcutaneous oxygen pressure (tcpO_2_), leg circumferences, and pain sensation were obtained at three time points: prior to therapy initiation (t0), on the first day following intermittent pneumatic impulse compression (IIC) (t1), and after IIC treatment conclusion on day 5 (t2). Measurements were consistently performed within the first hour following IIC treatment cessation. The tcpO_2_ measurement was conducted on the dorsal aspect of both feet, with results recorded after 20 min. The measurement was carried out in the department of vascular and endovascular surgery. Standard measuring tapes were utilized to assess ankle circumference, lower leg circumference (10 cm below the knee), and thigh circumference (15 cm above the knee). Additionally, patients reported pain sensation experienced in each leg using the Numeric Rating Scale (NRS 0-10). A blinded study physician or doctoral candidate conducted the measurements. The primary endpoint was defined as the reduction in leg circumference, while secondary endpoints comprised the improvement in tcpO_2_ and pain perception.

### 2.6. Wound-QoL

The Wound-QoL (Wound Quality of Life) Tool is a standardized questionnaire designed to assess the impact of chronic wounds on a patient’s quality of life. It is extensively utilized in clinical practice and research to evaluate patient-reported outcomes and inform treatment decisions. The instrument facilitates the measurement of physical, psychological, and social burdens associated with chronic wounds, including pain, mobility restrictions, and emotional distress across 17 evaluated questions each scaled from 0 to 4. Therefore, an overall score ranging from 0 to 68 can be obtained.

### 2.7. Sample Size Estimation

For our study, we aimed to recruit participants over a two-year period. Based on prior case volume at our institution regarding stasis dermatitis and experienced treatment courses, we initially estimated a sample size of approximately 20 patients who would meet the inclusion criteria for this exploratory study. However, over the course of the study, the final number of enrolled patients was 17. The COVID-19 pandemic may have influenced patient recruitment, as hospital admissions and treatment patterns were affected during this period. Our prospective approach ensured systematic data collection and adherence to predefined clinical parameters, allowing us to obtain a representative sample for analyzing the effects of IIC in this patient population.

### 2.8. Statistical Analysis

All data were compiled in Excel^®^ (Microsoft Corporation, Redmond, WA, USA). Mean values and standard deviations (SDs) or medians and ranges were calculated as appropriate, and two-sided paired *t*-tests were conducted to compare the intervention and control groups. A *p*-value < 0.05 was considered statistically significant. Analysis was performed using GraphPad Prism^®^ (Version 10.2.2 GraphPad Software Inc., La Jolla, CA, USA).

## 3. Results

### 3.1. Patient Demographics

A total of 17 patients, comprising 11 males and 6 females, with a mean age of 68 years, (median age 66, range 53–88) were enrolled in the study after obtaining informed consent. The mean body mass index (BMI) was 38.34 (median 37.4, range 22.6–65.1). On average, the patients in our cohort with a body mass index (BMI) exceeding 35 were classified as having grade II obesity. Table 2 presents the baseline patient characteristics in detail.

### 3.2. Clinical Course of Stasis Dermatitis Under Therapy

**Figure 4:**
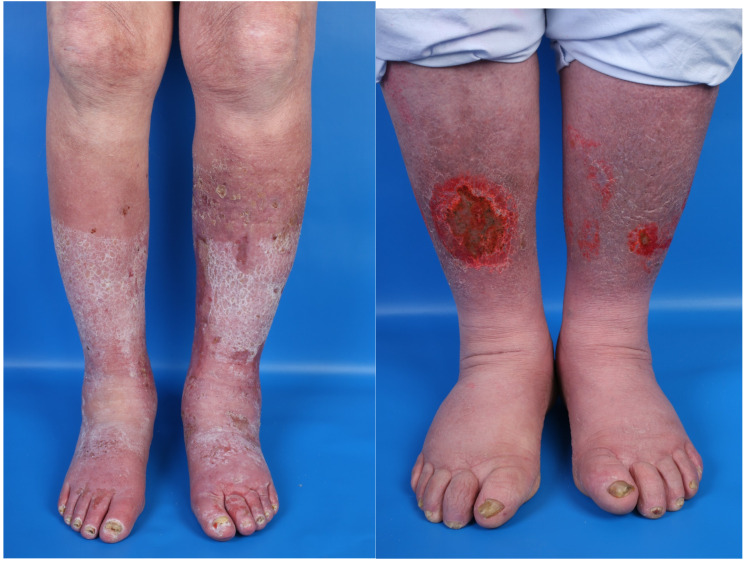
Clinical presentation of patient six on the left side and patient one on the right side during their inpatient stay. In the region of both lower extremities, erythematous plaques with scaling, erosions, and crusts are observed.

Ten patients presented with classic CVI as the etiology of their stasis dermatitis. Furthermore, lymphedema was identified as the causative factor in four patients who did not exhibit CVI as an underlying condition. One patient was diagnosed with high-grade but stable cardiac insufficiency, one patient presented with lipedema, and one patient was found to have an underlying arterial disease as the etiology of the stasis dermatitis. Stasis dermatitis manifested bilaterally in all patients. Figure 4 illustrates patients number 1 and 6 during their inpatient stay as representative examples. IIC demonstrated high tolerability, with only one patient withdrawing from the study due to non-compliance. This patient discontinued participation after the initial measurement, without experiencing pain or adverse effects.

### 3.3. Quality of Life

**Figure 5:**
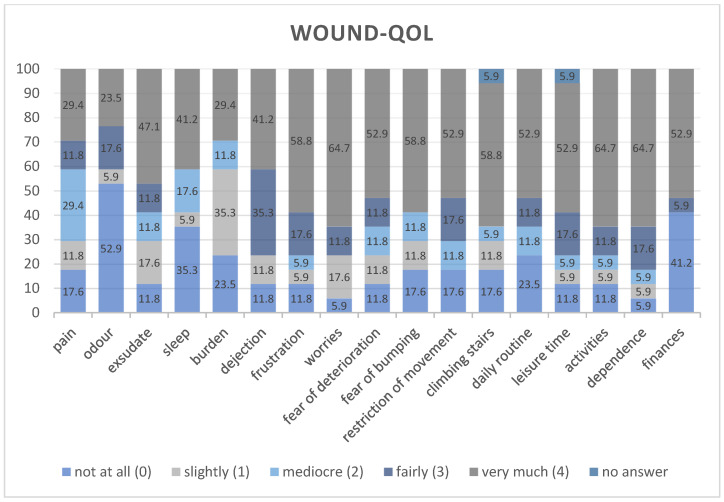
Wound-QoL of all patients.

The patients in this study exhibited significant burden due to their illness. The median quality-of-life score (Wound-QoL) was 50, with a mean value of 44.5 (range 0–68). Notably, aspects such as worry, dependence on others, and restrictions in activities were reported as particularly distressing. Figure 5 illustrates the Wound-QoL results for all patients.

### 3.4. Leg Circumferences (Ankle, Lower Leg, and Upper Leg)

**Figure 6:**
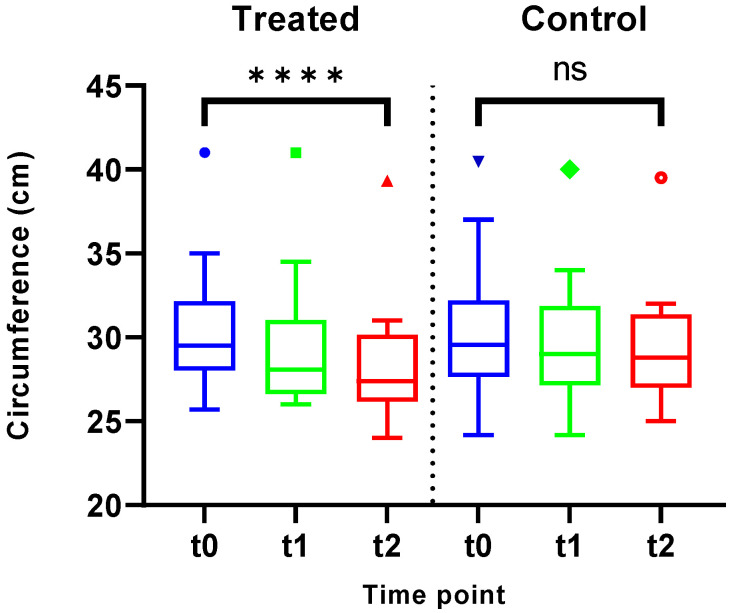
Analysis of the IIC-treated legs revealed a statistically significant reduction in leg circumference at the ankle region (*p* < 0.0001) over the course of the study. In contrast, the legs subjected to standard therapy alone exhibited no significant alterations in these parameters (*p* = 0.106). **** = significant; ns = not significant.

Figure 6 presents the results of ankle circumference measurements, and Table 3 presents all circumferences findings as an overview.

In IIC-treated legs, a significant reduction in ankle region circumference was observed (MD = −2.125 cm, SD = 1.593, *p* < 0.0001) over time. Legs receiving standard therapy alone demonstrated no significant change in these parameters over time (MD = −0.888 cm, SD = 2.067, *p* = 0.106,). Furthermore, lower leg circumferences exhibited improvement in the treatment group, surpassing standard therapy, albeit not reaching statistical significance (MD = −0.931 cm, SD = 1.881, *p* = 0.066 in the treatment group and MD = −0.000 cm, SD = 2.766, *p* = 0.100 in the control group). Leg circumferences above the knee (upper leg) did not demonstrate statistically significant improvement in either group (MD = 0.350 cm, SD = 1.881, *p* = 0.370 in the intervention and MD = −0.144 cm, SD = 1.194, *p* = 0.637 in the control group). Table 3 shows all outcome parameters of circumferences, oxygen saturation (tcpO_2_), and the level of pain with MD, SD, and *p*-value.

### 3.5. Tissue Oxygen Saturation (tcpO_2_)

**Figure 7:**
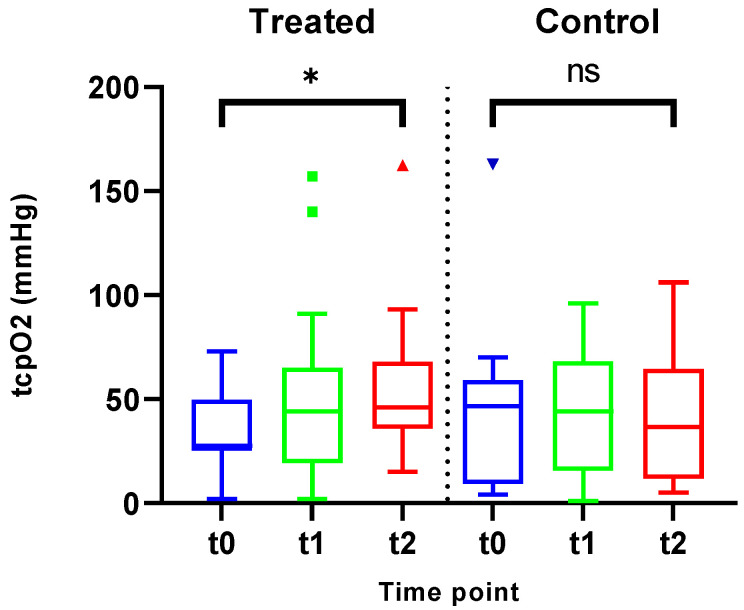
Analysis of the IIC-treated legs revealed a statistically significant improvement in tissue oxygen saturation (*p* = 0.012). The legs subjected to standard therapy alone exhibited no significant change in these parameters (*p* = 0.754). * = significant; ns = not significant.

IIC-treated legs exhibited a statistically significant improvement in tissue oxygen saturation (MD = 19.87 mmHg, SD = 27.82, *p* = 0.012) over time (Figure 7). Conversely, oxygen saturation decreased in the control group (MD = −2.375 mmHg, SD = 29.82 *p* = 0.754).

### 3.6. Level of Pain

No exacerbation of pain was observed under IIC therapy, particularly in patients with active lower leg ulcerations. There was no significant difference in pain comparing t0 with t2 in either group (MD = −0.313, SD = 1.662, *p* = 0.464 in the intervention group and MD = −0.375, SD = 2.247, *p* = 0.515 in the control group).

## 4. Discussion

Stasis dermatitis is increasingly observed in older dermatological patients, yet standardized treatment protocols remain lacking [7]. In many cases, inpatient management is required, placing significant demands on healthcare resources, particularly in the application of compression therapy. While stasis dermatitis is traditionally linked to CVI, our findings highlight that up to one-third of affected patients present with additional underlying conditions, suggesting a more complex pathophysiology than previously recognized. The condition imposes a substantial burden on patients, leading to pain, reduced mobility, and social isolation, as well as contributing to significant healthcare costs. The patients in our study had a poor quality of life with a mean Wound-QoL of 44.5, which represents a high burden of disease. These findings align with the review by Yosipovitch et al., which emphasizes the profound impact of stasis dermatitis on patients’ quality of life and the economic strain on healthcare systems [6]. The review also underscores the limitations of current management strategies, particularly patient adherence to compression therapy, and the absence of approved pharmacological treatments specifically targeting skin changes associated with stasis dermatitis.

Given these challenges, our study evaluated the integration of IIC into the clinical treatment algorithm for stasis dermatitis under real-world conditions. While IIC has been extensively studied in other medical contexts, its application in stasis dermatitis remains unexplored despite its potential benefits [13].

IIC is a therapeutic method based on the principles of controlled impulse pressure application, stimulating venous return and microcirculation. In our study, we demonstrated that ankle edema in patients with stasis dermatitis could be significantly reduced with an additional IIC if consistent therapy was provided for five days compared to standard treatment alone. Elastocompressive wrapping alone, which constituted the standard therapy of edema management, did demonstrate an improvement but no significant change over time and proved significantly less effective in comparison to additional IIC treatment. In routine clinical practice, it has been generally assumed that the daily application of a properly administered compression bandage results in a progressive reduction in lower leg edema [14,15]. In our study, the compression bandages were applied by trained personnel. Considering these findings, the application of compression bandages by patients themselves or by untrained personnel warrants even greater scrutiny [16].

A small-scale study in the field of chronic venous leg ulcers supports our conclusion. This investigation, conducted by Arcelus et al. on nine patients with CVI, demonstrated that the combination of IIC for two hours daily with support stockings resulted in a significant reduction in venous residual volume fraction [17]. Furthermore, based on patient-reported outcomes, a significant decrease in edema and pain was observed, and the foot compression devices were well tolerated by all study participants.

In our investigation, we were unable to replicate this significant edema reduction in the calf and thigh region. A mean reduction in lower leg circumference was observed in the treatment group compared to the control group. However, the difference was not statistically significant. Edema reduction through impulse compression has also been demonstrated in several trauma surgery studies, with a primary focus on the ankle region. For instance, one study by Caschman et al. has demonstrated that IIC has a significant benefit in the management of adults with isolated ankle fractures who were unsuitable for immediate open reduction and internal fixation [18].

Furthermore, our study indicated that the oxygen supply to the tissue also improved significantly with impulse compression therapy, which was not observed with wrapping alone. Eberlein et al. also demonstrated that IIC treatment induces a decrease in pH value in chronic venous leg ulcers when the affected legs were treated with impulse compression, which was attributed to an increased microcirculation [19]. tcpO_2_ is an indicator of improved tissue oxygenation. The underlying mechanisms driving this effect are multifactorial and subject to ongoing investigation.

One hypothesis attributes the rise in tcpO_2_ to the reduction in edema, which alleviates interstitial pressure, enhances microcirculatory flow, and subsequently improves oxygen diffusion to the tissues. However, emerging evidence suggests that the increase in tcpO_2_ may also be directly related to shear stress-induced nitric oxide (NO) release. The repetitive mechanical impulses generated by IIC stimulate endothelial cells, promoting NO synthesis, which leads to vasodilation and improved perfusion independent of edema reduction [5]. It is probable that both mechanisms contribute synergistically: edema reduction optimizes the physical environment for oxygen exchange, while NO-mediated vasodilation enhances microvascular blood flow. The measured tcpO_2_ value prior to impulse compression may have been low due to the presence of existing edema, which could have impaired oxygen diffusion to the skin surface. This represents a potential limitation of this study, as the reduction in edema following impulse compression may have contributed to an apparent increase in tcpO_2_, independent of actual improvements in microvascular perfusion. Whether there is a direct effect on the microcirculation or whether the increase in the tcpO2 value in our study group is only a secondary effect cannot ultimately be fully clarified. Consequently, the observed changes in tcpO_2_ should be interpreted with caution, considering the confounding effect of edema on baseline measurements. A further limitation of our proof-of-concept study is the relatively small number of patients, which must be considered when interpreting the results. Furthermore, the treatment is not associated with pain even for patients with acute stasis dermatitis and accompanying ulcerations or erosions. It is necessary to consider the design of our study, which did not investigate the use of IIC against placebo alone, as observed in numerous other studies in the traumatological field. In our study setting, we investigated IIC against the standard of care as an additional treatment modality. As previously noted, this approach is advocated by many researchers in this clinical context. Consequently, the measurable effects may be somewhat attenuated. Nevertheless, we observed a significant positive effect on edema reduction and oxygen saturation. The initial concerns regarding pain exacerbation and potential adverse effects in the application of IIC in stasis dermatitis were not substantiated in this proof-of-principle study.

Intermittent pneumatic compression (IPC) and intermittent pneumatic impulse compression (IIC) are both therapeutic techniques used to improve circulation and reduce edema. While IPC applies a steady, rhythmic pressure to the affected limb, alternating between compression and relaxation phases, IIC uses pulsating pressure in brief, intense bursts, followed by rest periods. This difference in pressure application may make IIC more effective for stimulating localized blood and lymphatic flow, potentially leading to faster tissue healing. A direct comparison in the form of a comparative study on the effectiveness of the two compression systems has not yet been carried out.

Our findings provide evidence supporting the use of IIC as an adjunct therapy, addressing both the inflammatory and circulatory components of stasis dermatitis. Further research is warranted to confirm its clinical efficacy and establish its role in routine dermatological practice.

## 5. Conclusions

Our study provides substantial evidence that IIC serves as a valuable adjunctive therapy for stasis dermatitis, which is associated with a high reduction in the quality of life of those affected. This research demonstrates that IIC significantly reduces ankle edema and improves tissue oxygenation, surpassing the efficacy of compression bandages alone. IIC presents a promising alternative that enhances treatment outcomes without additional patient burden. Moreover, the results align with existing research in other medical fields, supporting the hypothesis that IIC-induced improvements in oxygenation stem from both edema reduction and endothelial stimulation via nitric oxide release. Notably, no exacerbation of pain was observed, even in patients with acute stasis dermatitis and ulcerations, reinforcing the safety and tolerability of IIC in this patient population.

Despite these promising results, further large-scale clinical trials are necessary to corroborate these findings and optimize treatment protocols.

## Figures and Tables

**Table 1:** Inclusion and exclusion criteria.

Inclusion Criteria	Exclusion Criteria
Patients aged between 30–90 years	Patient aged under 30 and over 90 years
Written informed consent	Absence of a written declaration of consent
Diagnosis of stasis dermatitis	Acute untreated serious infection on affected extremity
	Acute decompensated cardiac insufficiency
	Acute phlebitis (inflammation of the veins) Acute thrombosis or pulmonary embolism

**Table 2:** Patient characteristics.

Characteristic	n = 17
Age (years)	
Mean (SD)	68.12 (11.27)
Gender, n (%)	
Male	11 (64.71)
Female	6 (35.29)
BMI	
Mean (SD)	38.34 (12.88)
Wound-QoL	
Mean (SD)	44.47 (17.50)

SD, standard deviation.

**Table 3:** Outcome parameters of all patients.

	MD	SD	*p*-Value
Circumferences (cm)			
Ancle			
Treated	−2.125	1.593	<0.0001
Control	−0.888	2.067	0.106
Lower leg			
Treated	−0.931	1.881	0.066
Control	0.000	2.766	0.100
Upper leg			
Treated	−0.350	1.516	0.370
Control	−0.144	1.194	0.637
Oxygen saturation (tcpO_2_, mmHg)			
Treated	19.87	27.82	0.012
Control	−2.375	29.82	0.754
Pain (NRS)			
Treated	−0.313	1.662	0.464
Control	−0.375	2.247	0.515

MD, mean difference; SD, standard deviation.

## Data Availability

The raw data supporting the conclusions of this article will be made available by the authors on request.

## Abbreviations

The following abbreviations are used in this manuscript:

CVI	Chronic venous insufficiency
DOAJ	Directory of open-access journals
IIC	Intermittent pneumatic impulse compression
IPC	Intermittent pneumatic compression
MD	Mean difference
MDPI	Multidisciplinary Digital Publishing Institute
SD	Standard deviation
SOC	Standard of care
tcpO_2_	Transcutaneous oxygen pressure
QoL	Quality of life
VEGF	Vascular endothelial growth factor
